# Noise-Induced Hearing Loss Awareness and Prevention: A Cross-Sectional Study Among the Population of the Southern Region of Saudi Arabia

**DOI:** 10.7759/cureus.66793

**Published:** 2024-08-13

**Authors:** Rajab A Alzahrani, Wafaa S Taishan, Mujtaba Ali, Abdulrahman A Almaymoni, Turki S Althunayyan, Ibrahim N Al Sulaiman, Assal B Hobani, Ziad A Ibrahim, Manar O Alharbi, Thamer Alzahrani, Jad M Mony, Yassmin Aljedaani

**Affiliations:** 1 Unit of Otolaryngology, Department of Surgery, Faculty of Medicine, Al Baha University, Al Baha, SAU; 2 Department of Otolaryngology, King Faisal Hospital, Makkah, SAU; 3 Department of Surgery, Faculty of Medicine, Al Baha University, Al Baha, SAU; 4 College of Medicine and Surgery, King Khalid University, Abha, SAU; 5 Department of Medicine and Surgery, Qassim University, Qassim, SAU; 6 College of Medicine, Najran University, Najran, SAU; 7 Faculty of Medicine, Ibn Sina National College for Medical Studies, Jeddah, SAU; 8 Department of Family Medicine, King Abdulaziz University Hospital, Jeddah, SAU; 9 College of Medicine, King Abdulaziz University, Jeddah, SAU; 10 Department of Psychiatry, King Saud University, Riyadh, SAU; 11 Department of Otology/Neurotology, King Fahad General Hospital, Jeddah, SAU

**Keywords:** prevalence, saudi arabia, prevention, awareness, noise-induced hearing loss

## Abstract

Background: Noise-induced hearing loss (NIHL) is a prevalent and preventable health issue globally. This study aims to evaluate the symptoms, knowledge, beliefs, and preventive practices regarding NIHL among the general population of the southern region of Saudi Arabia.

Materials & methods: A cross-sectional study was conducted from May to July 2024, using a self-administered, validated electronic questionnaire distributed in Arabic via social media platforms. The questionnaire assessed socio-demographic data, NIHL awareness, attitudes toward prevention, and personal practices regarding noise exposure. The sample included 400 participants analyzed using SPSS version 23 (IBM Corp., Armonk, NY), with associations measured through the chi-square and Fisher’s exact tests.

Results: Most participants were Saudi nationals (97%) and females (81.3%). Symptoms of NIHL, such as tinnitus and the need to increase TV or radio volume, were prevalent among participants. Most participants (88.5%) were aware that high sound levels affect hearing, yet only 9.5% correctly identified the minimum duration of exposure that could harm hearing. Social media was the primary source of information (51.3%). Positive preventive practices were noted, with 66% lowering device volumes and 55.3% recommending sound restrictions on tablets. Significant associations were found between better preventive practices and higher income as well as marital status.

Conclusion: The study highlights the high basic awareness of NIHL but identifies critical knowledge gaps regarding the minimum sound level and duration that affect hearing negatively. Enhanced public health education and technological interventions are needed to improve prevention practices. Future research should include longitudinal studies and diverse populations to better understand and address NIHL.

## Introduction

Noise-induced hearing loss (NIHL) has been a major disability affecting society globally and it is one of the most common forms of acquired hearing impairment. Globally, over 5% of the world's population - or 430 million people - requires rehabilitation to address their disabling hearing loss (including 34 million children) [1]. While locally, the prevalence of NIHL was 22%, 51.8%, and 26% in different cities in Saudi Arabia [2-4].

Disabling hearing loss can have varying consequences on the quality of life depending on its severity. It can lead to social isolation since it affects communication with family and friends, causes difficulties at work, and has an influence on emotional and economic aspects of life [5-7]. There are many factors contributing to hearing loss, one of which is exposure to a high-intensity level of sound for extended periods of time, which is known to cause NIHL. It is considered the second most common cause of sensorineural hearing loss, following age-related hearing loss, despite the fact that it is almost totally avoidable [8].

NIHL is linked to impairment of speech intelligibility over loud noise, persistent hearing threshold shift development, and injury to the inner ear's sensory hair cells [9]. Reversible hearing loss can occur from exposure to noise up to 60 dB for more than 60 minutes, whereas permanent hearing loss develops from exposure to noise at 85 dB for at least eight hours per day [10].

Numerous health problems, both auditory and non-auditory, can result from excessive noise exposure. It is a significant preventable cause of irreversible hearing loss worldwide. Additionally, the body may experience psychological, physiological, and biochemical alterations as a result of such exposure [11].

In this study, we aimed to evaluate the symptoms of hearing impairment, explore the knowledge and beliefs regarding NIHL, and assess the participants' attitudes toward prevention.

## Materials and methods

This was a cross-sectional study conducted from May 1st to July 10, 2024, to evaluate the symptoms of hearing impairment and explore the knowledge and beliefs regarding NIHL among the population of the southern region of Saudi Arabia. Both Saudi and non-Saudi people living in the southern region of Saudi Arabia and aged between 18 and 80 years, who agreed to participate in the current survey, were included in the study, and populations who do not live in the southern region of Saudi Arabia and aged under 18 or above 80 years, who did not agree to participate were excluded.

The study was approved by the Institutional Research Board of Al Baha University (approval number: REC/SUR/BU-FM/2024/70). The participants were informed about the study's aims and assured of data confidentiality, and consent was obtained from each participant before participating in the study.

Sample size

The estimated sample size according to Cochran's equation is about 385, with a precision level of ±5% and confidence level of 95%. The study enlisted 400 participants.

Sampling frame

Data were collected by using an anonymous self-administered, reliable, and validated electronic questionnaire and it was modified to meet the study objectives. The questionnaire was translated into Arabic and then distributed among the general population of the southern region of Saudi Arabia through social platforms such as WhatsApp and Telegram. All participants were informed in detail about the study's aims and data confidentiality. The questionnaire required consent from the participants to participate in this study. The questionnaire had two versions in English and Arabic languages, therefore the participants had a right to choose the language out of the two, whichever they felt comfortable with. The questionnaire was composed of socio-demographic data, evaluated population awareness of NIHL, and explored issues and attitudes around it.

Statistical analysis plan

For data analysis, all questions were coded into a spreadsheet and transferred into SPSS version 23.0 (IBM Corp., Armonk, NY). Data were presented as frequencies and percentages. A common grading method was used for questions of knowledge about noise-inducing hearing loss as follows: one point was given to the correct option, zero for the incorrect answer, and for neutral, a participant who correctly answered more than 50% of the questions (i.e., scored more than five points out of 10) was considered as having good knowledge about NIHL. For practice questions, one point was given to the positive option, and zero for the negative option, and the participant who correctly answered more than 50% of the questions (i.e., scored five points or more out of nine) was considered as having positive practice toward NIHL. The association between variables was measured using the chi-square test and Fisher's exact test. A significant p-value was set below 0.05.

## Results

A total of 400 participants from the southern region of Saudi Arabia were included in this study. The vast majority were females (n = 325, 81.3%) and Saudi nationals (n = 388, 97%) (Table 1). Regarding their age, most of them aged between 18 and 25 years (34.5%), 46 and 55 years (26.5%), and 36 and 45 years (23%). About half of the participants (51.8%) were married at the time of the study and 164 (41%) were single. Regarding their educational level, more than two-thirds (70.5%) had a university degree, and 83 (20.8%) had a high school education. More than a third of the participants (35.8%) were from Abha and about a third of them (33.5%) were from Jazan. Most of the participants had an income of less than 5,000 Saudi riyal (SAR) (44%). The rest had an income of 5,000 to 10,000 SAR (21.8%), 10,001 to 15,000 SAR (18%), 15,001 to 20,000 SAR (13%), and more than 20,000 SAR (3.3%).

**Table 1:** Socio-demographic characteristics of the participants (n = 400). N: number; %: percentage.

Variable	Categories	N (%)
Gender	Male	75 (18.8)
	Female	325 (81.3)
Age (years)	18-25	138 (34.5)
	26-35	55 (13.8)
	36-45	92 (23)
	46-55	106 (26.5)
	>55	9 (2.3)
Nationality	Saudi	388 (97)
	Non-Saudi	12 (3)
Marital status	Single	164 (41)
	Married	207 (51.8)
	Divorced	21 (5.3)
	Widower	8 (2)
Educational level	Primary school or less	6 (1.5)
	High school level	83 (20.8)
	University level	282 (70.5)
	Postgraduate level	29 (7.3)
Region/residency	Abha	143 (35.8)
	Al-Baha	45 (11.3)
	Jazan	134 (33.5)
	Najran	78 (19.5)
Average monthly income (Saudi riyal)	<5,000	176 (44)
	5,000-10,000	87 (21.8)
	10,001-15,000	72 (18)
	15,001-20,000	52 (13)
	>20,000	13 (3.3)

**Figure 1:**
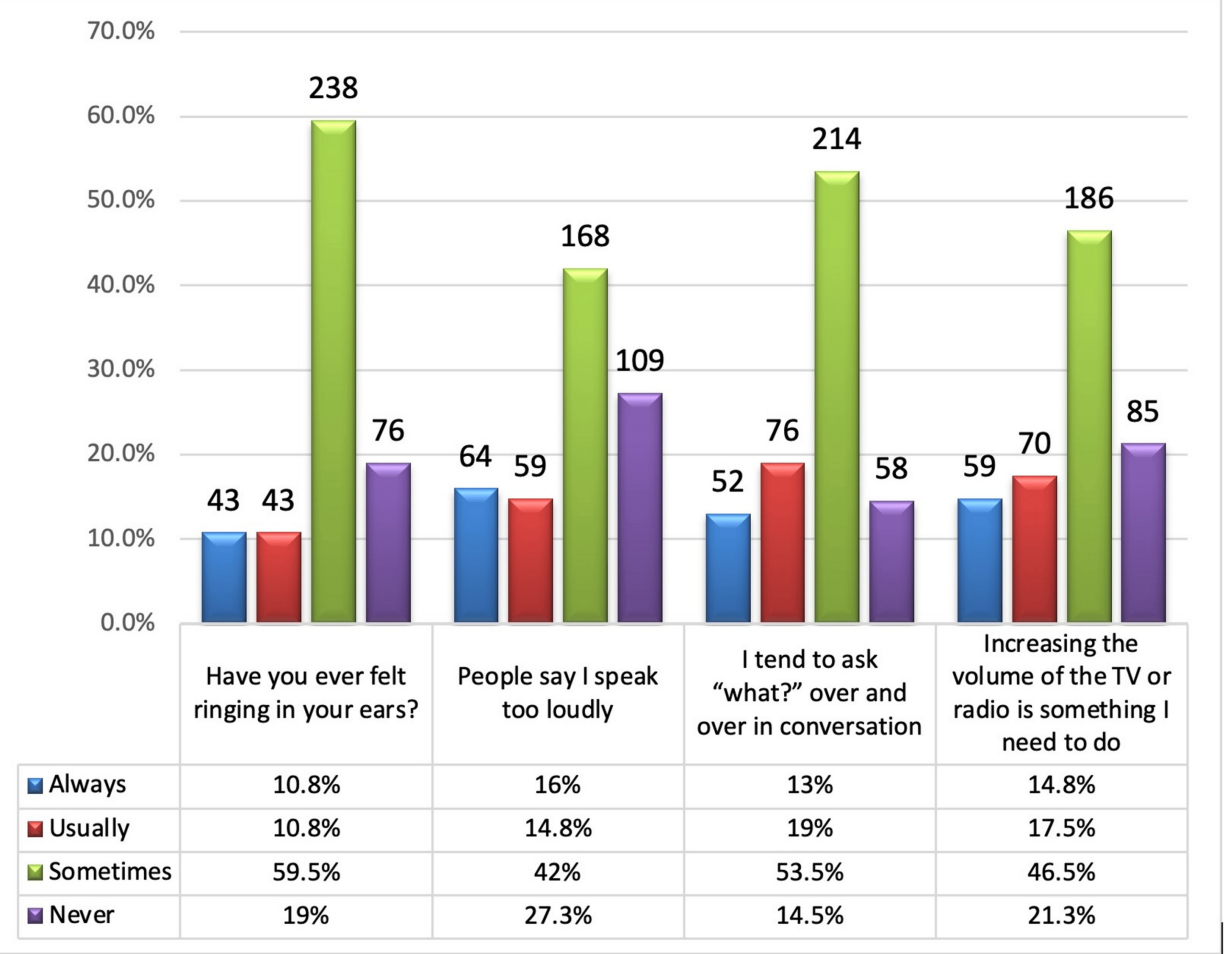
Signs and symptoms related to noise-induced hearing loss.

Regarding participants' signs and symptoms related to NIHL, the results in Figure 1 showed that ringing in the ears was reported by 81% with more than half of the participants hearing it sometimes (59.5%). About 168 (42%) of the participants reported that “sometimes people said I talk loud” and 214 (53.5%) reported that they sometimes tend to ask “what?” over and over in conversation. Increasing the volume of the TV or radio is always necessary for 78.7% of the participants with about 186 (46.5%) needing it sometimes. The average time needed by the participants to adapt to environmental sound when exposed to loud noise was 2.9 ± 3.25 hours, almost two-thirds of the participants (n = 268, 67%) said that they needed an hour to adapt to environmental sound, and 93 (23.3%) needed five hours, 30 (7.5%) needed 10 hours, and nine (2.3%) needed 15 hours for adaptation.

Regarding the participants' beliefs and knowledge about NIHL, the majority (88.5%) of the participants correctly knew that hearing is affected by high sound levels and 338 (84.5%) correctly answered that their hearing could be affected if they live or work in a noisy environment (Table 2). About 310 (77.5%) of the participants said that hearing loss can worsen by listening to loud sounds. Concerning signs of hearing loss, 161 (40.3%) of the participants correctly knew that hearing low or muffled sounds during everyday conversation indicates early signs of hearing loss and only 103 (25.8%) knew that the feeling of ringing in the ear is a sign of hearing loss. More than half (54.8%) correctly knew that hearing loss is indicated by frequent loudness of the TV or radio and more than two-thirds (72.3%) knew that noise-induced hearing problems can be prevented. Moreover, 179 (44.8%) of the participants think that they currently have enough information about how exposure to loud noise can harm their hearing while 121 (30.3%) did not.

**Table 2:** Beliefs and knowledge about noise-induced hearing loss. N: number; %: percentage; *: correct answer.

Question	Yes	No	I don't know
	N (%)		
Do high sound levels affect hearing?	354 (88.5)*	14 (3.5)	32 (8)
Does living or working in a noisy environment affect your hearing?	338 (84.5)*	25 (6.3)	37 (9.3)
Can hearing loss worsen when listening to loud sounds?	310 (77.5)*	24 (6)	66 (16.5)
Does hearing low or muffled sounds during everyday conversation indicate early signs of hearing loss?	161 (40.3)*	63 (15.8)	176 (44)
Is the feeling of ringing in the ear a sign of hearing loss?	103 (25.8)*	73 (18.3)	224 (56)
Does frequent loudness of the TV/radio indicate hearing loss?	219 (54.8)*	86 (21.5)	95 (23.8)
Can hearing problems caused by noise be prevented?	289 (72.3)*	18 (4.5)	93 (23.3)
Do I currently have enough information about how exposure to loud noise can harm my hearing?	179 (44.8)*	121 (30.3)	100 (25)

**Figure 2:**
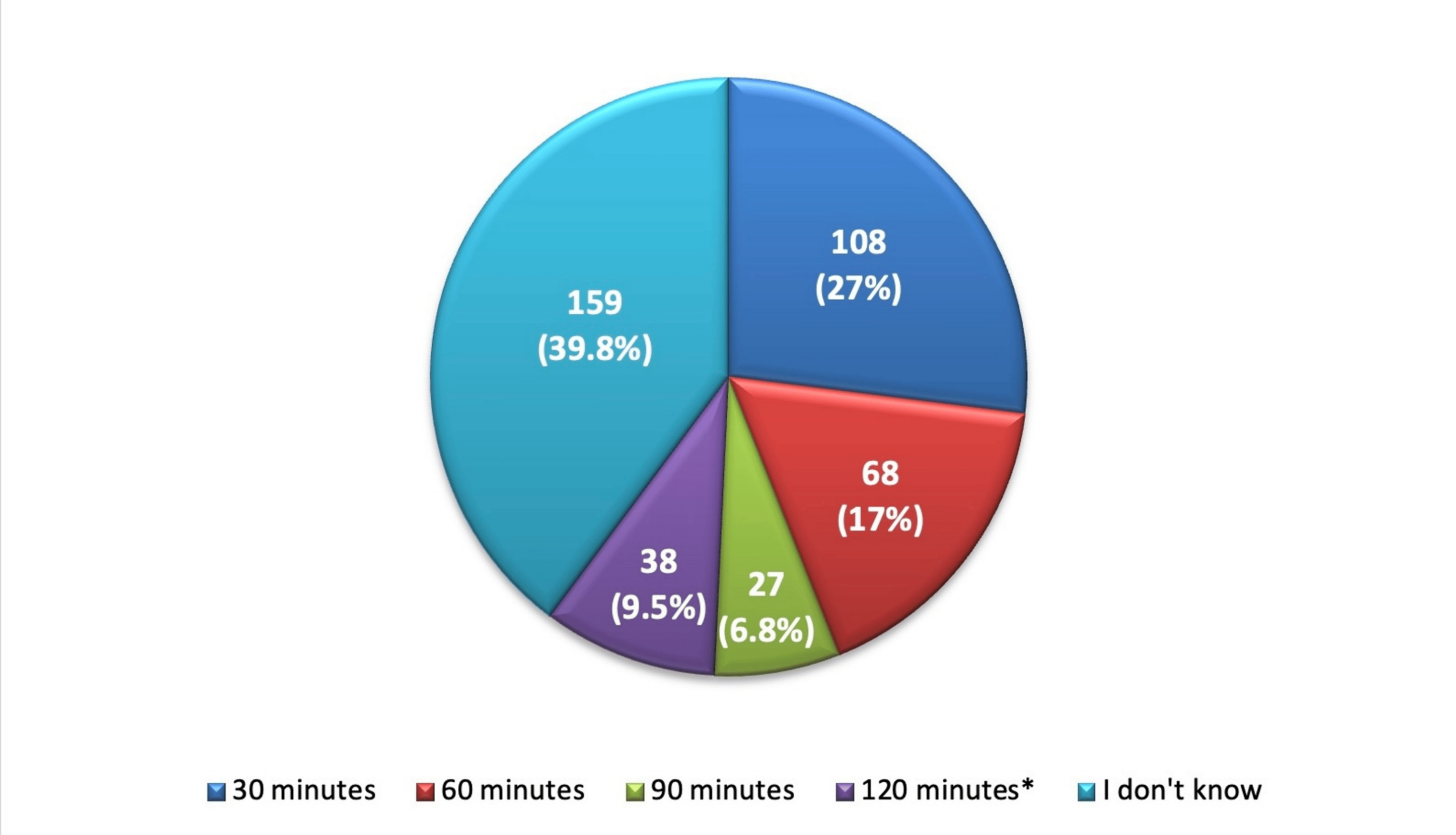
The minimum duration of listening to a loud noise source that may negatively affect hearing. *: correct answer.

When the participants were asked about the minimum duration of listening to a loud noise source that may negatively affect hearing, only 38 (9.5%) of them correctly knew that it is 120 minutes while nearly a third of them (27%) thought that it is 30 minutes and about 159 (39.8%) had no idea about the minimum duration (Figure 2).

**Figure 3:**
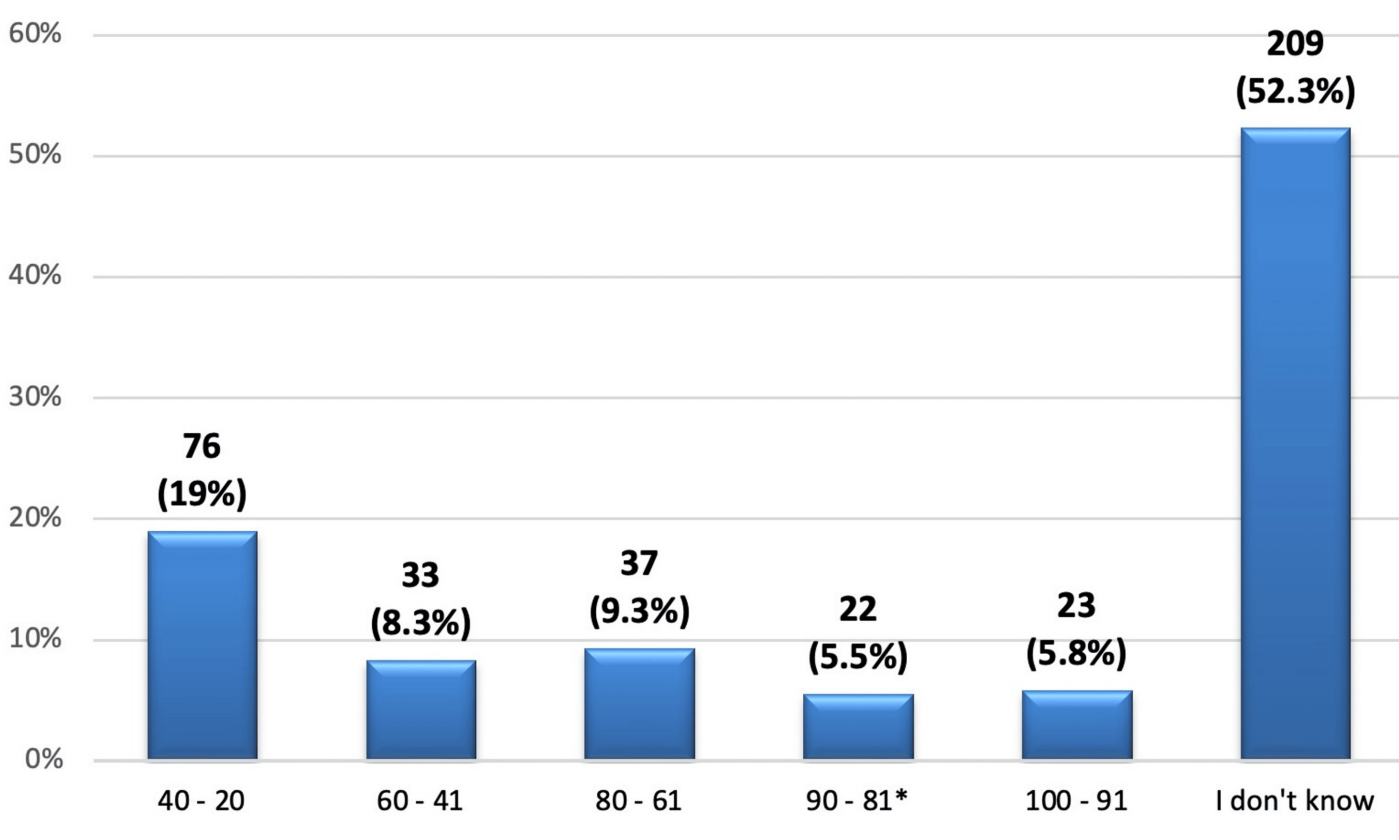
The minimum sound level that can negatively affect hearing (%). *: correct answer.

Concerning the minimum sound level that can negatively affect hearing, Figure 3 shows only 22 (5.5%) of the participants correctly knew that it is 81-90% while 76 (19%) think that it is 20-40%, and more than half of them (52.3%) did not know about the minimum sound level that can negatively affect hearing.

Regarding the practices and attitudes toward NIHL, most of the participants received their knowledge about it through social media (51.3%), followed by educational campaigns (18.3%) (Table 3). Almost two-thirds of the participants (66%) preferred to lower their device volume during the total listening time and about 221 (55.3%) recommended installing sound restriction on their tablet. Half of the participants (50%) were always ready to change their behavior if they heard or saw evidence that loud noise or sound levels affect their hearing. Most of the participants (91.5%) recommended placing warning indicators on audio devices to limit sound levels and 182 (45.5%) always prefer to use software to determine sound levels for themselves and their families.

**Table 3:** Sources of information, practices, and attitudes toward NIHL. N: number; %: percentage; NIHL: noise-induced hearing loss.

Question:	Categories	N (%)
The source of information that is commonly accessed about NIHL.	Educational campaigns	73 (18.3)
	Hospitals	49 (12.3)
	Schools & workplaces	49 (12.3)
	Shopping Center	7 (1.8)
	Social media	205 (51.3)
	Television	17 (4.3)
Do I prefer to lower my device volume during the total listening time?	Yes	264 (66)
	No	74 (18.5)
	I don't know	62 (15.5)
Recommend that the factory installs sound restrictions on my tablet.	Yes	221 (55.3)
	No	95 (23.8)
	I don't know	84 (21)
I am willing to change my behavior if I hear or see evidence that loud noise/sound levels affect my hearing.	Always	200 (50)
	Usually	89 (22.3)
	Sometimes	85 (21.3)
	Never	26 (6.5)
I recommend placing warning indicators on audio devices to limit sound levels.	Yes	366 (91.5)
	No	34 (8.5)
I prefer to use software to determine sound levels for myself and my family.	Always	182 (45.5)
	Usually	62 (15.5)
	Sometimes	99 (24.8)
	Never	57 (14.3)

The average score of knowledge about NIHL was 5.0 ± 1.96 (range: 0- 9) out of 10 and the median score was 5.0 (IQR: 4-6.75). About 175 (43.8%) had a good level of knowledge while 225 (56.3%) had poor knowledge. Regarding the participants' practices toward NIHL, the mean practice score was 6.2 ± 2.20 (range: 0-9) out of 9 and the median score was 7.0 (IQR: 5-8). More than two-thirds (n = 307, 76.8%) had positive practice toward NIHL while 93 (23.3%) had negative practice. Concerning the factors associated with knowledge and practice toward NIHL, there were no significant associations between knowledge and all sociodemographic factors (p > 0.05), whereas there was a significant association between practice and marital status (p = 0.034); married participants had the highest level of practice compared to others (Table 4). Also, there was a significant association between practice and average monthly income (p = 0.011), and the level of practice seemed to improve with increasing income. Other factors did not significantly associate with level of practice toward NIHL (p > 0.05).

**Table 4:** Factors associated with knowledge and practice toward NIHL. N: number; %: percentage; C: chi-square test; F: Fisher's exact test; *: significant p-value; NIHL: noise-induced hearing loss.

Variable	Level of knowledge		P-value	Level of practice		P-value
	Good	Poor		Positive	Negative	
	N (%)			N (%)		
Gender:						
Male	29 (38.7)	46 (61.3)	0.325^C^	56 (74.7)	19 (25.3)	0.636^C^
Female	146 (44.9)	179 (55.1)		251 (77.2)	74 (22.8)	
Age (in years):						
18-25	53 (38.4)	85 (61.6)	0.096^C^	97 (70.3)	41 (29.7)	0.073^C^
26-35	21 (38.2)	34 (61.8)		39 (70.9)	16 (29.1)	
36-45	43 (46.7)	49 (53.3)		75 (81.5)	17 (18.5)	
46-55	56 (52.8)	50 (47.2)		89 (84)	17 (16)	
>55	2 (22.2)	7 (77.8)		7 (77.8)	2 (22.2)	
Nationality:						
Saudi	170 (43.8)	218 (56.2)	0.883^C^	299 (77.1)	89 (22.9)	0.485^F^
Non-Saudi	5 (41.7)	7 (58.3)		8 (66.7)	4 (33.3)	
Marital status:						
Single	61 (37.2)	103 (62.8)	0.133^F^	116 (70.7)	48 (29.3)	0.034^F^*
Married	102 (49.3)	105 (50.7)		171 (82.6)	36 (17.4)	
Divorced	9 (42.9)	12 (57.1)		15 (71.4)	6 (28.6)	
Widower	3 (37.5)	5 (62.5)		5 (62.5)	3 (37.5)	
Educational level:						
Primary school or less	2 (33.3)	4 (66.7)	0.762^F^	4 (66.7)	2 (33.3)	0.862^F^
High school level	34 (41)	49 (59)		65 (78.3)	18 (21.7)	
University level	128 (45.4)	154 (54.6)		217 (77)	65 (23)	
Postgraduate level	11 (37.9)	18 (62.1)		21 (72.4)	8 (27.6)	
Average monthly income (Saudi riyal):						
<5,000	71 (40.3)	105 (59.7)	0.056^C^	122 (69.3)	54 (30.7)	0.011^C^*
5,000-10,000	33 (37.9)	54 (62.1)		67 (77)	20 (23)	
10,001-15,000	34 (47.2)	38 (52.8)		62 (86.1)	10 (13.9)	
15,001-20,000	32 (61.5)	20 (38.5)		46 (88.5)	6 (11.5)	
>20,000	5 (38.5)	8 (61.5)		10 (76.9)	3 (23.1)	

## Discussion

Due to exposure to loud sounds, millions of individuals worldwide suffer from some degree of hearing impairment, making NIHL a serious public health problem. Since this kind of hearing loss is completely avoidable, it is even more crucial to inform people about the precautions they may take to safeguard their hearing [12]. This study is aimed at exploring the awareness and prevention regarding NIHL among the Saudi population of the southern region.

The findings of this study contribute important information toward understanding the prevalence of symptoms related to NIHL within a sample of residents from the southern region of Saudi Arabia. Tinnitus, speaking loudly, requiring repetition in conversations, and turning up the volume on any devices were found to be some clear signs of the condition. Most participants (81%) complained of tinnitus, which is a sign that is commonly associated with NIHL. This is higher compared to other regional studies like Haji et al. [5] (2022), where lower tinnitus prevalence was established among the Saudi population. Such high prevalence also correlates with Batts et al.'s [6] (2024) study, where it was found that tinnitus is present in many people who work or live in noisy environments. The study found that 42% of the participants complained that they sometimes speak loudly. This symptom can be related to hearing problems since people tend to raise their voices due to the lack of sound stimuli [13-15]. Our findings are consistent with similar findings in another study that showed that the intensity of noise increases vocal volume in people who are exposed to it [16]. The fact that 53.5% of participants said that they often say “what” during the conversation points to the problems that people with NIHL face when trying to understand speech in conditions accompanied by noise. This symptom is in line with the difficulties in speech discrimination that have been described in the literature [17]. This concurs with Kim et al. [10] (2022), who noted that a significant proportion of patients with NIHL present with speech comprehension difficulties.

Notably, turning the volume up on the TV or radio was cited as a crucial necessity by 78.7% of the respondents. This finding also corroborates the findings of Moore et al. [11] (2022), who observed that in NIHL patients, louder sounds are perceived clearly. The mean time taken to adapt to environmental sound after exposure to loud noise was found to be 2.9 ± 3.25 hours. This long period of adaptation shows the long-term negative impact of noise exposure on the human auditory system [18-20]. A similar observation was made by a study that discussed residual hearing damage in people who are exposed to high levels of noise [21]. An hour of adaptation required by 67% of participants is evidence of a rather long recovery, which corresponds to the delayed auditory recovery revealed in some studies [20].

According to the results of our study, it can be stated that the awareness and knowledge of the participants about NIHL are quite high. A majority of the participants provided accurate information on the causes, signs, and measures of NIHL. Such a high level of knowledge is required for the development and maintenance of proper hearing conservation programs [22]. A considerable number of the participants (88.5%) showed good knowledge of the adverse effects of high sound levels and most participants considered noise risks at the basic level, which is consistent with the findings reported in a Jordanian study conducted among a similar group of workers [23]. However, only 25.8% of our participants reported tinnitus (ringing in the ears) as a sign of hearing loss, which is significantly lower than respondents found in an urban population study [13].

In addition, 77.5% of the participants agreed with the statement that hearing loss could become worse when exposed to loud sounds. This is in line with Alzahrani et al. [24] (2024), who called for awareness of the fact that NIHL is a gradual process. Understanding that hearing loss may get worse with prolonged exposure to loud sounds is vital for encouraging protective actions [24]. Regarding the perception of early symptoms of hearing impairment, 40.3% of respondents recognize that low or muffled tones during normal conversations are symptoms of hearing loss. Yet, only 25.8% identified tinnitus or ringing in the ears as a sign of hearing loss. This divergence leaves a gap in specific knowledge about the observable signs of NIHL. As noted by Haji et al. (2022) in a study, tinnitus has been identified as a significant early sign of NIHL, and more efforts should be made to raise public awareness of this sign [5].

With regard to primary prevention, results revealed that 72.3% of participants agreed that NIHL can be prevented, which indicates a favorable attitude toward potential preventive measures. This is slightly higher than the findings by Basheer et al. (2019), where they noted a 60% awareness rate of the preventability of NIHL among industrial workers [25]. This difference could be attributed to the fact that our sample has a higher level of education compared to other studies. However, 44.8% of participants perceived that they had sufficient information about how exposure to loud noise affects their hearing while 30.3% of them did not. This means that although people may have minimum knowledge regarding NIHL, they require more information about the disease and the precautionary measures to be taken [11,24]. Our study stresses the need for increasing public awareness as a way of addressing these knowledge gaps and strengthening preventive practices [22].

Lack of knowledge was also observed in relation to the minimum duration and decibel level that may have a detrimental effect on the hearing ability of individuals, of which only 9.5% of participants expressed their understanding that 120 minutes of exposure to loud noise could potentially harm their hearing. This was lower than what has been reported in other studies conducted in different areas whereby there is high awareness of the disease most probably due to constant health promotion campaigns [26]. When asked about the decibel level that has a chance of causing hearing damage, 5.5% of the participants chose the correct range of 81-90. More than half (52.3%) of the participants were unaware of the minimum sound level, while 19% of the participants assumed the minimum sound level to be between 20 and 40 decibels. This is quite alarming as people may not be aware of the dangers associated with these sound levels that are heard in their environments. The WHO states that frequencies above 85 dBA (A-weighted decibels) for prolonged periods may cause hearing loss [27]. The lack of correct responses in certain aspects points to a significant lack of health literacy in this area. Earlier research has also revealed that when there are increased public health campaigns and awareness programs, awareness regarding NIHL and its prevention improves [28].

In the present study, the primary source of information on NIHL for most participants was social media (51.3%), followed by educational campaigns (18.3%). This aligns with recent studies highlighting the growing influence of social media in disseminating health information [29]. A substantial 66% of participants reported preferring to lower the volume of their devices during listening sessions, reflecting a proactive approach to protecting their hearing. This practice is consistent with recommendations from the WHO that advocate for limiting the volume and duration of exposure to loud sounds to prevent NIHL [27]. Additionally, 55.3% of participants recommended installing sound restrictions on their tablets, indicating a desire for technological solutions to aid in hearing conservation. The willingness to adopt new behaviors based on evidence of hearing damage (50% always ready to change) is encouraging and implies a potential receptivity to public health interventions that could further educate and encourage protective behaviors against NIHL [20,28]. Moreover, the strong support for warning indicators on audio devices (91.5%) indicates a broad consensus on the need for more aggressive preventive measures, mirroring trends in public health policies advocated in studies from the US and Europe, which call for manufacturers to integrate more health warnings on entertainment and communication devices [19,22].

The analysis of factors associated with knowledge and practice revealed no significant associations between knowledge levels and sociodemographic factors. However, there were significant associations between practice and marital status (p = 0.034), as well as average monthly income (p = 0.011). Married participants and those with higher incomes exhibited better practices toward preventing NIHL. This may be attributed to increased awareness and access to resources that facilitate protective behaviors among these groups. The significant association between income and health behaviors is not a novelty in the literature and is explained by enhanced access to information and health-promoting settings [2,22].

There are some limitations in this study. First, the cross-sectional design compromises on generalizing causality between knowledge, attitudes, and practices toward NIHL. Secondly, the questionnaire data collected relies on self-reporting, which can be subject to response bias, and participants who completed the electronic questionnaire may not be representative of the general population because of limited internet connectivity or technology literacy. However, the sampling technique that involved the use of social media to disseminate the survey may not have captured a random sample of the target population, especially those who rarely or never use social media.

## Conclusions

This study highlights the prevalence of NIHL symptoms among the southern Saudi population and reveals substantial awareness about the condition's risks and preventive measures. Despite this, significant knowledge gaps regarding the specific sound levels and exposure durations that can cause hearing damage remain. Social media, as the primary information source, presents an opportunity for targeted, accurate public health messaging. Future efforts should focus on enhancing NIHL education through social platforms, incorporating technological solutions like sound level warnings on devices, and integrating hearing health education into schools and workplaces. Longitudinal research is necessary to assess the impact of these interventions on improving NIHL knowledge and practices over time.

## Appendices

Questionnaire

Demographic information

1. Gender:

- Male

- Female

2. Age:

- 18-25

- 26-35

- 36-45

- 46-55

- 56 and above

3. Marital status:

- Single

- Married

- Divorced

- Widowed

4. Nationality:

- Saudi

- Non-Saudi

5. Educational level:

- Primary or less

- High school level

- University level

- Postgraduate level

6. Monthly income:

- Less than 5,000 SAR

- 5,000-10,000 SAR

- 10,001-15,000 SAR

- 15,001-20,000 SAR

- Above 20,000 SAR

7. From which region are you from:

- Abha

- Jazan

- Najran

- Al Baha

Evaluate the symptoms of hearing impairment:

1. Ringing in the ears:

- Always

- Usually

- Sometimes

- Never

2. People said I talk loud:

- Always

- Usually

- Sometimes

- Never

3. I tend to ask “What ?” repeatedly in a conversation:

- Always

- Usually

- Sometimes

- Never

4. Increasing the volume of the TV or radio is something I do:

- Always

- Usually

- Sometimes

- Never

5. Time I need to adapt to surrounding environmental sound when exposed to loudness (hour):

- 1

- 5

- 10

- 15

Knowledge and beliefs regarding NIHL:

1. Do high volume levels affect hearing?

- Yes

- No

- I don’t know

2. Does living or working in a noisy environment affect hearing?

- Yes

- No

- I don’t know

3. Hearing impairment could get worse by listening to loud sounds?

- Yes

- No

- I don’t know

4. Does the hearing of low/muffled voices during daily conversation indicate the early signs of hearing impairment?

- Yes

- No

- I don’t know

5. Is the sensation of ringing in the ear a sign of hearing impairment?

- Yes

- No

- I don’t know

6. Does the frequent increase in TV/radio volume indicate a sign of hearing impairment?

- Yes

- No

- I don’t know

7. Are noise-induced hearing problems preventable?

- Yes

- No

- I don’t know

8. Do I currently have enough information concerning the danger posed by exposure to loud noises on hearing ability?

- Yes

- No

- I don’t know

9. The minimum duration of listening to a loud noise source that could negatively affect one’s hearing is:

- 30 minutes

- 1 hour

- 1 ½ hours

- 2 hours and more

- I don’t know

10. The minimum volume level that could negatively affect hearing is (%):

- 20-40

- 41-60

- 61-80

- 81-90

- 91-100

- I don’t know

Protective measures to stop NIHL

1. Typically accessed source of information about NIHL:

- Educational campaigns

- Commercial centers

- Schools and job settings

- Hospitals

- Social media

- TV

2. Do I prefer to decrease the volume of my device over the total time of listening?

- Yes

- No

- I don’t know

3. I recommend that the factory should install a voice-limiting feature on my device:

- Yes

- No

- I don’t know

4. I’m ready to change my behavior if I hear or see evidence that suggests that loud noise/sound levels affect hearing:

- Always

- Usually

- Sometimes

- Never

5. I recommend putting warning indicators on audio devices to limit volume levels:

- Yes

- No

6. I prefer using a program to limit sound levels for me and my family:

- Always

- Usually

- Sometimes

- Never
